# Chiral Pt/ZrO_2 _Catalysts. Enantioselective Hydrogenation of 1-phenyl-1,2-propanedione 

**DOI:** 10.3390/molecules15053428

**Published:** 2010-05-12

**Authors:** Claudia Urbina, Cristian Campos, Gina Pecchi, Carmen Claver, Patricio Reyes

**Affiliations:** 1 Facultad de Ciencias Químicas, Universidad de Concepción, Casilla 160-C, Concepción, Chile; E-Mails: curbina@udec.cl (C.U.); gpecchi@udec.cl (G.P.); ccampos@udec.cl (C.C.); 2 Departamento Química Física i Inorgànica, Universitat Virgili i Rovira, Marcel.lí Domingo, s/n. 43007 Tarragona, Spain; E-Mail: carmen.claver@urv.cat (C.C.)

**Keywords:** colloidal platinum, mesostructured ZrO_2_, enantioselective hydrogenation, chiral modifier

## Abstract

The enantioselective hydrogenation of 1-phenyl-1,2-propanedione over Pt colloids stabilized with (*R,S*)-4,5-dihydro-4,5-diphenyl-2-(6-cyanopyridinyl)imidazoline (CI) supported on a meso-structured ZrO_2_ under a pressure of 40 bar of H_2_ at 298 K has been investigated_._ The metal loading in all catalysts was 1 wt%. The effect of the amount of chiral modifier on the metal particle size and on the catalytic behavior was analyzed. It was found that as the CI/Pt molar ratio increases from 2.5 to 3.5 the Pt crystal size decreases from 3.0 to 1.8 nm. All catalysts were very active in the studied reaction, with the most active one being the catalyst with smaller Pt particles, whereas the selectivity is higher in those catalysts with larger chiral modified Pt metal particles.

## 1. Introduction

In the last few years, the applications of single enantiomers of chiral compounds have increased significantly, especially for their uses in the field of pharmaceuticals, agrochemicals, and flavors and fragrances [1]. Even though homogeneous metal complexes with chiral ligands are the most widely used catalysts, heterogeneous catalysis has emerged as an important alternative since Orito *et al* [2] reported the enantioselective hydrogenation with ee’s up to 95% of ethyl pyruvate to *(R)*-ethyl lactate over cinchonidine-modified Pt catalysts, Other research teams have evaluated these catalytic systems in the mentioned reaction [3,4] using Pt, Pd and Ir-supported on SiO_2_, Al_2_O_3_ and others [5,6,7,8,9]. Indeed, this reaction is presently a sort of test reaction in the field of asymmetric catalysis.

However, the reaction becomes more difficult, both in terms of enantio- and regioselectivity (rs), when the substrate has more than one center to be hydrogenated, such as during dione hydrogenation in 1-phenyl-1,2-propanedione [10,11,12]. As far as regioselectivity is concerned, the product of interest is *(R)*-1-phenyl-1-hydroxy-2-propanone, which has interesting applications in the synthesis of compounds used as precursors for hypertension and asthma treatments, among others [13,14].

Supported platinum catalysts have been widely studied for this type of applications [15], and it has been found that metal particle size plays an important role in asymmetric synthesis when performed in the presence of cinchonidine as a chiral modifier (CM). The most promising catalytic behavior has been obtained for catalysts with a metal particle size around 3.0 nm and metal loading close to 5 wt % . The catalysts containing lower metal loadings usually display smaller particle sizes, and therefore are inappropriate for this purpose. However, using colloidal preparation procedures, metallic crystals with different average particle sizes can be prepared. Thus, even with low metal loading, catalysts with higher metal metal particle size can be obtained. In fact, it is well known that metal colloids with different particle sizes can be prepared by this procedure in the presence of a chiral modifier, which may lead to a preferential hydrogenation to *(R)*-ethyl lactate during the hydrogenation of ethyl pyruvate [16,17]. Metal crystals stabilized with chiral complexes used as catalysts in different reactions have been widely reported [18,19,20,21,22,23].

Support selection is relevant in the preparation of heterogeneous catalysts for the enantioselective hydrogenation of α-ketoesters, α-functionalized ketones and α,β-diketones [24]. Several groups have tried to improve the hydrogenation of ethyl pyruvate by preparing cinchonidine-modified Pt catalysts on various supports. Hall *et al*. [25] described the use of a Pt/MCM-41 catalyst that exhibits low reaction rates due to mass transfer limitations and enantiomeric excess up to 64%. Zeolites of different structures and acidities can also display highly enantioselective hydrogenation supported on Pt catalysts. In fact, Böhmer *et al*. [26] have reported the re-use of Pt/HNaY zeolite in the hydrogenation of ethyl pyruvate in acetic acid and cyclohexane reaching a high e.e. (88%). Additionally, e.e. up to 75% was reported for Pt/clay catalysts (28% e.e. for Rh/K-10) [27], while e.e.s below 35% were described for Pt/C catalysts, despite improvement by oxidative treatment [28]. In the last few years, research has focused on improving the support’s structural and morphological properties to enhance the catalytic behavior [29,30,31]. One of the main advantages of these structured solids compared to the traditional supports is that they can produce a confinement effect that can favorably affect both activity and the selectivity in different reactions.

Thus, the use of Pt on mesoporous MCM-41 in the enantioselective hydrogenation of 1-phenyl-1,2-propanedione was successfully demonstrated. Toukoniitty *et al*. [29] have studied three different Pt metal contents (5, 10 and 15 wt % ) on MCM-41 modified with (-)-cinchonidine (CD), obtaining a maximum enantiomeric excess of (*R*)-1-phenyl-1-hydroxy-2-propanone close to 44% using the 15 wt % Pt/MCM-41 catalyst. This behaviour was attributed to the larger metal particle size displayed by this catalyst. Reyes *et al.* [30] reported similar results in terms of activity and enantioselectivity using a 1 wt % Pt/MCM-41, which is more active than a 1 wt%Pt/SiO_2_ catalyst with comparable metal particle size. The observed behavior was explained in terms of the confinement effects produced in the MCM-41 hexagonal channels. Thus, ZrO_2 _has shown important properties from the catalytic point of view and ZrO_2 _films on a metal surface have also been extensively used as a protection agent against corrosion [31]. Oxide gels in the presence of a surfactant or appropriate templates produce nanotubes, and thus ZrO_2_ is mesoestructured when prepared from a surfactant, such as cetyltrimethylammonium bromide (CTMABr), which acts as template for nanotubes with a given channel diameter [32]. ZrO_2 _nanotubes may also be obtained by covering carbon nanotubes with oxide gels followed by calcination [33]. These solids display high surface areas and are materials with potential catalytic applications [34]. Two types of chiral modifiers have been widely studied: tartaric acid used as modifier of Ni catalysts [35] and cinchona alkaloid and analogs, where cinchonidine (CD) [36,37] is the most representative example. However, other chiral ligands may also display interesting behavior.

**Figure 1:**
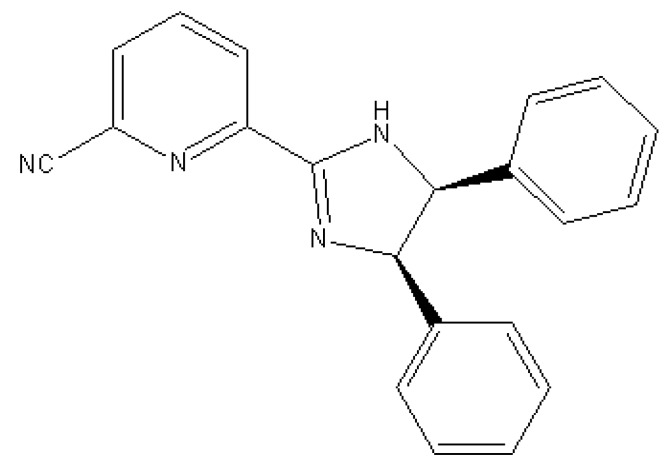
Structure of (*R,S*)-4,5-dihydro-4,5-diphenyl-2-(6-cyanopyridyl)imidazole.

In the present work, we have prepared (*R,S*)-4,5-dihydro-4,5-diphenyl-2-(6-cyanopyridyl)imidazole, which is an imidazoline derivative used as a chiral modifier (see Figure 1). This ligand has been used by Claver *et al*. [38,39] to prepare a Pd complex with applications in fine chemistry.

**Figure 2:**
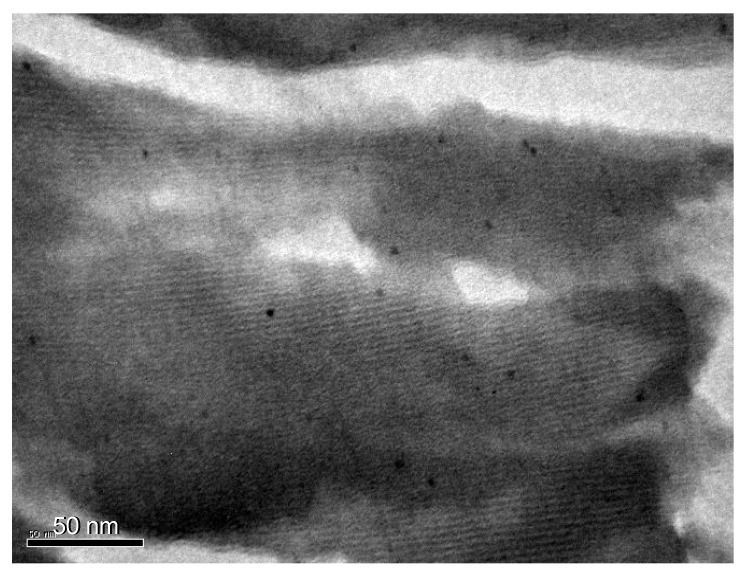
.Transmission electron micrographs of Pt(2.5)/CNTsZrO_2_ catalysts.

In this work, Pt-supported catalysts in which the active phase was obtained from a colloidal dispersion, followed by impregnation on nanostructured ZrO_2_ support were synthesized, [33,34]. The colloid dispersion was prepared by reduction of a Pt precursor in the presence of the previously mentioned ligand used as chiral modifier and also as stabilizer of the colloid, because it limits the excessive growing of the metal nanoparticles. The prepared catalysts were characterized and evaluated in the batch hydrogenation of 1-phenyl-1,2-propanedione (Figure 2) at 298 K and 40 bar of H_2_ using cyclohexane as solvent. 

## 2. Results and Discussion

### 2.1. Characterization

X-ray diffraction patterns of the prepared catalysts do not show any diffraction line characteristic of monoclinic and tetragonal phases and no low angle XRD reflections. Similar results have been found by Larsen *et al.* [39] and Blin *et al.* [40], who described the synthesis of sulfated mesoporous zirconia mediated by lauryl sulfate in alkaline solution and CTMABr, respectively. In contrast to the synthesis of silica mesoporous structured materials, these authors suggest that the pore formation mechanism does not imply detectable bi- or three-dimensional X-ray ordering. Indeed, even if the low angle signal is not observed in the XRD-pattern, samples exhibit a uniform pore size distribution in the mesoporous range. In the present study, in agreement with Blin [40], the absence of a small angle reflection line can be attributed to a relatively large pore size distribution. Additionally, the absence of a diffraction line due to Pt may be explained by the small size of some metal nanoparticles (shown by TEM analysis)

The nitrogen adsorption-desorption isotherms correspond to type IV in the BDDT classification [41]. The isotherm’s adsorption branch can be decomposed in three parts: the monolayer-multiple adsorption of nitrogen, the capillary condensation of nitrogen within the mesopores, and then saturation. The hysteresis loops reveal the presence of open capillaries characteristic of MCM-41 type of materials. However, in the CNTsZrO_2_, the pore size distribution is centered in the range 2.5 to 4 nm and display a BET surface area of 87 m^2^ g^-1^ and a pore volume of 0.18 cc g^-1^. 

TEM micrographs of the calcined ZrO_2_ materials show, for the Pt/CNTsZrO_2 _samples, typical shapes of nanostructured solids with a regular hexagonal array of mesoporous channels, results that are in good agreement with those previously reported by Beck *et al*. [42] as can be seen in Figure 2. 

However, the study of different areas of the samples with TEM resulted in the detection of amorphous areas in the solid. The presence of Pt crystals located inside the channel of structured supports can be clearly observed, with a very narrow metal crystal distribution, in the range 1 to 4 nm. As the ligand/Pt molar ratio increases, the average decreases from 3.0 to 1.8 nm. Using a same preparation procedure, if no ligand is added during the reduction of platinum precursor, the colloid is not stable; therefore as comparison, in absence of modifier, the catalyst was prepared by impregnation of chloroplatinic acid followed by calcinations at 300 ºC and reduction in hydrogen at 500 ºC. The particle size was 3.8 nm. A summary of the characteristic of the studied catalysts is given in Table 1.

**Table 1:** Platinum particle size(d) obtained by TEM and metal dispersion (D) evaluated from TEM results of Pt(x)/CNTsZrO_2 _catalysts.

Catalysts	d_TEM_ , nm	D_TEM_
Pt(0.0)/CNTsZrO_2_	3.8	0.24
Pt(2.5)/CNTsZrO_2_	1.8	0.51
Pt(3.0)/CNTsZrO_2_	2.6	0.35
Pt(3.5)/CNTsZrO_2_	3.0	0.31

### 2.2. Catalytic activity in 1-phenyl-1,2-propanedione hydrogenation

**Figure 3:**
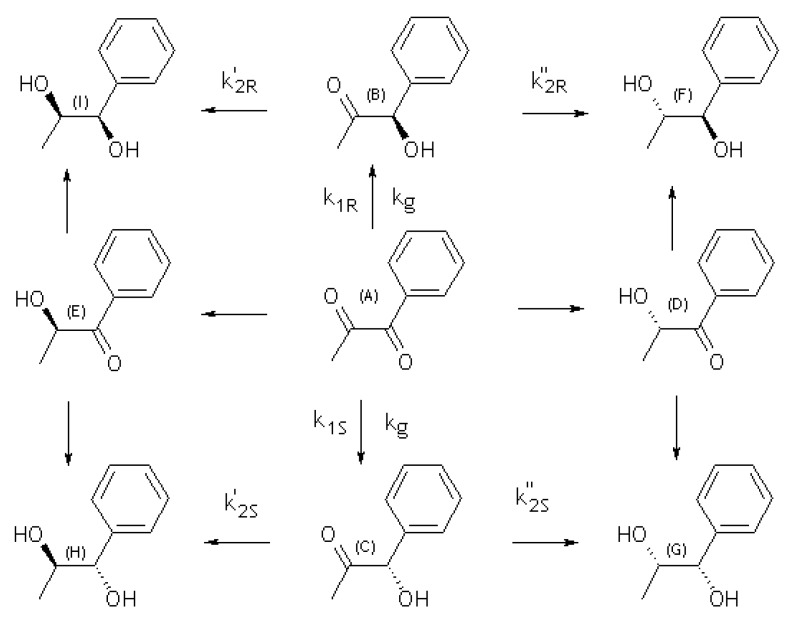
Reaction scheme of the hydrogenation of (**A**) 1-phenyl-1,2-propanedione. (**B**) (*R*)-1-hydroxy-1-phenyl-2-propanone; (**C**) (*S*)-1-hydroxy-1.phenyl-2-propanone; (**D**) (*S*)-2-hydroxy-1-phenyl-1-propanone; (**E**) (*R*)-2-hydroxy-1-phenyl-1-propanone; (**F**) (1*R*,2*S*)-1-phenyl-1,2-propanediol; (**G**) (1*S*,2*S*)-1-phenyl-1,2-propanediol; (**H**) (1*S*,2*R*)-1-phenyl-1,2-propanediol; (**I**) (1*R*,2*R*)-1-phenyl-1,2-propanediol.

Figure 3 shows a scheme of the enantioselective hydrogenation of 1-phenyl-1,2-propanedione. The reaction was studied at 25 ºC and 40 bar of H_2 _on zirconia-supported modified-Pt catalysts*.*

Using these catalysts, it was found that a preferential formation of *(R)*-1-phenyl-1-hydroxy-2-propanone can be induced. The evolution of the conversion level over time for the studied catalysts present similar trends for all catalysts and are compatible with a pseudo first-order reaction, showing differences in activity depending to metal loading, where the regioisomers *(R)* and *(S)*-1-hydroxy-2-phenylpropanone are the main products.

**Figure 4:**
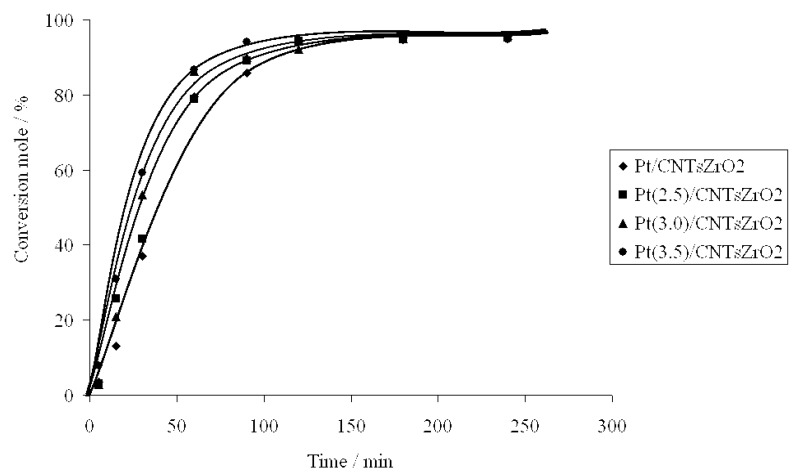
Evolution of the conversion level with time during 1-phenyl-1,2-propanodione hydrogenation on Pt/CNTsZrO_2_ catalysts.

This behavior may be explained mainly in terms of electronic factors. The aromatic ring contributes to polarization to a greater extent the C=O bond close to the ring, and therefore the reactions lead to (**B**) (*R*)-1-hydroxy-1-phenyl-2-propanone and (**C**) (*S*)-1-hydroxy-1.phenyl-2-propanone, whereas the regioisomers (**D**) (*S*)-2-hydroxy-1-phenyl-1-propanone and (**E**) (*R*)-2-hydroxy-1-phenyl-1-propanone are not favored.

**Figure 5:**
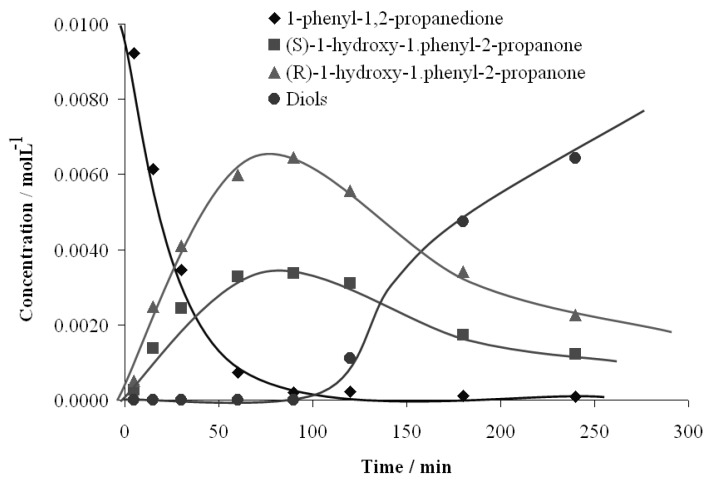
Evolution of the concentration of the substrate and different reaction products during 1-phenyl-1, 2-propanodione hydrogenation. T = 25 ºC, P H_2_= 40 bar, catalyst Pt(3.0)/CNTsZrO_2_ weight 100 mg and CD concentration = 0.25 mM.

Figure 5 shows the kinetics of products formation in a batch reactor in the 1-phenyl-1,2-propanedione hydrogenation on one representative catalyst, Pt (3.0) /CNTsZrO_2_ catalyst. The results indicate that under the experimental conditions, the catalyst displays a high activity, with a conversion level close to 90% at 1,800 min, moderate ee (30%) and complete regioselectivity. The evolution of the dione concentration and the other reaction products follow the expected trends: the dione concentration decreases through a first-order law, leading to hydroxyketones, (*R*)-1-hydroxy-1-phenylpropanone and (*S*)-1-hydroxy-(*R*)-1-phenylpropanone as intermediate compounds (**B** and **C** in Figure 3). A maximum reaction time of close to 125 min is obtained for the most active catalyst (Pt/CNTsZrO_2_). A longer time to reach a maximum hydroxyketone concentration was observed for the other catalysts: 105 and 150 min for Pt/MSZrO_2_ and Pt/ZrO_2 _respectively (not shown). All studied catalysts were completely regioselective; no products generated by the hydrogenation of the C=O bond located far from the aromatic ring were obtained: (*R*)-2-hydroxy-1-phenylpropanone and (*S*)-2-hydroxy-1-phenylpropanone (**E** and **D**, respectively, in Figure 3). In all cases, the presence of over-hydrogenated diol products was observed, and the evolution of the diol concentrations follow the expected sigmoid curve, characteristic of final products of consecutive reactions. Thus, the reaction pathway may be represented by irreversible, parallel and consecutive reactions. 

The first-order reaction rate constants, denoted as k_g_ for dione consumption, can be easily obtained from log C_Ao/_C_A_* vs.* t plots. The reaction constants k_1R_ and k_1S_, corresponding to the formation of (*R*)- 1-hydroxy-1-phenylpropanone and (*S*)-1-hydroxy-(*R*)-1-phenylpropanone, may be obtained from the global first order constant and the ratio of the concentration of these compounds (**B** and **C** in the scheme), that is C_B_/C_S._ Additionally, the reaction constants k_2R _and k_2S_, associated to the consecutive reactions, can be evaluated from the well-known equations for intermediates of consecutive reactions, in terms of C_max_ and t_max_, and are the maximum concentration and the time required to get that level of the corresponding intermediates, respectively. The obtained results are summarized in Table 2.

The rate constants for the first and second hydrogenation steps (k_1 _and k_2_) increase as the molar ratio ligand/Pt increases. This can be explained by the fact that the catalyst prepared in the presence of higher ligand/Pt molar ratio possesses higher metal dispersion and consequently higher hydrogenation rates. In all cases, the confinement effect produced by the support provides an enhancement in the reaction rate compared to a non-structured support [15]. The presence of the chiral ligand on the catalyst surface favored the pathway to produce the *R*-intermediate (k_1R_ > k_1S_); in the second step, the hydrogenation of the *S*-intermediate also improved (k_2S _> k_2R_).

**Figure 6:**
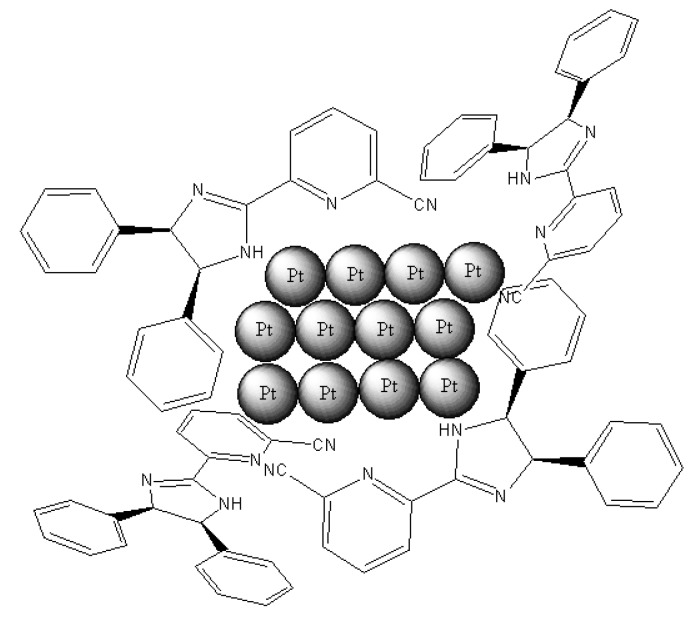
Interaction diagram Pt with (*R,S*)-4,5-dihydro-4,5-diphenyl-2-(6-cyano-pyridyl)imidazole in the stabilization of metal nanoparticles.

The reaction performed in absence of ligand (on the catalyst) does not show enantiomeric enhancement, confirming that a chiral molecule is necessary for the generation of modified catalytic sites responsible of the production of a given enantiomer. The enantiomeric excess displayed by the catalysts does not significantly change with the conversion level, although it is affected by the ligand/Pt molar ratio used in the preparation of the catalysts. As this ratio increases from 2.5 to 3.5, the Pt particle size decreases. Consequently, the ligand remaining linked to the metal surface also increases but not in the right position. Therefore, the platinum surface should lie parallel to the aromatic ring, leading to a decrease in the ee (see Figure 6). 

**Table 2:** First order reaction rate constants, in the hydrogenation of 1-phenyl-1, 2-propanodione at 298 K on Pt(x)/CNTsZrO_2 _catalysts.

Catalyst	k_1g_ /10^2^± 0.2 10^2^ min^-1^	k_1R_ /10^2^± 0.2 10^2^ min^-1^	k_1S_ /10^2^± 0.2 10^2^ min^-1^	k_2R_ /10^2^± 0.2 10^2^ min^-1^	k_2S _/10^2^± 0.2 10^2^ min^-1^
Pt(0.0)/CNTsZrO_2_	4.4	2.2	2.2	0.98	0.98
Pt(2.5)/CNTsZrO_2_	4.7	2.8	1.9	0.44	1.02
Pt(3.0/CNTsZrO_2_	5.2	3.3	1.9	0.59	1.13
Pt(3.5)/CNTsZrO_2_	6.0	4.0	2.0	0.60	1.10

k_2R_ = k'_2R _+ k''_2R _; k_2S_ = k '_2S _+ k''_2S_

Even though the obtained ee values are moderate, which confirms the difficulty of this reaction to induce chirality, they are not too far from the best reported values for enantioselectivity in the same reaction [7,8,9,10,11,12]. The fact that the three modified catalysts display almost no change in the ee values for the hydrogenation of 1-phenyl-1,2-propanodione, close to 30%, may be understood, considering that, apart from an optimum modifier concentration, the catalysts require a given metal particle size.

The increases in the rate constant for the first and second hydrogenation steps (k_1 _and k_2_) with the ligand /Pt molar ratio may be attributed to two different effects: i) the catalyst prepared in the presence of higher ligand/Pt molar ratio possesses higher metal dispersion and consequently higher hydrogenation rates and ii) the presence of a higher proportion of ligand anchored to metallic sites in the catalysts occurred to a greater extent in the modified sites, which are even more active than the metallic sites. In all cases, the confinement effect produced by the support enhances the reaction rate in comparison with a non-structured support [26]. The presence of the chiral ligand on the catalyst surface favored the pathway that produces the *R*-intermediate (k_1R_ > k_1S_); in the second step, the hydrogenation of the *S*-intermediate is also improved (k_2S _> k_2R_).

When the reaction is performed in absence of a chiral modifier on the catalyst surface, the reaction takes place without enantiomeric enhancement, and therefore CM contributes to the generation of modified catalytic sites that are enantioselective and more active than the metallic sites. 

## 3. Experimental

### 3.1. Support preparation

Carbon nanotubes were prepared following a procedure already reported [43]. Carbon nanotubes were mixed with an aqueous solution of ZrO(NO)_3_. This suspension was kept under ultrasonic vibrations for 10 min and then under boiling conditions for 3 h to evaporate the solvent. The final solid was calcined in Ar at 600 ºC for 2 h. A grey powder was obtained and was then calcined at 600 ºC in air for 2 h to remove the CNTs, resulting in a white powder. This support was labeled as CNTsZrO_2_.

### 3.2. Synthesis of the chiral ligand

The chiral pyridinylimidazoline ligand, (*R,S*)-4,5-dihydro-4,5-diphenyl-2-(6-cyanopyridyl)-imidazoline was prepared from 2,6-dicyanopyridine (0.77 mol), which was treated with meso-1,2- diphenylendiamine (0.77 mol) in chlorobenzene (5 mL) in the presence of ytterbium(III) trifluoromethanesulfonate [Yb(OTf)_3_, 0.14 mole]. The mixture was stirred for 24 h under reflux. The product was characterized by ^1^H- and ^13^C-NMR in a multinuclear Bruker AC instrument at 200/50 MHz using CDCl_3_ as solvent. The same signals and chemical shifts reported by Claver *et al.* were found [38]. 

### 3.3. Catalysts preparation

Colloidal Pt was prepared as follows. An aqueous solution containing 0.60 mmole of H_2_PtCl_2_ dissolved in 160 mL of water was heated to 100 ºC, then a solution of the 0.1 mol L^-1^ ligand in HCOOH was added, and reflux maintained for 60 min The amount of this later solution was varied according the desired ligand/Pt molar ratio. The solution shows initially a yellow color; due to the reduction of Pt (IV) to Pt(0), the solution became dark, becoming black after 10 min. Once the colloidal dispersion was obtained, it was slowly cooled down to room temperature, washed with a saturated solution of NaHCO_3_ (100 g L^-1^) and filtered. The amount of ligand added during the preparation of colloidal platinum to stabilize the Pt-colloid ligand with ligand/Pt molar ratios of 0, 2.0, 3.0 and 3.5 respectively. Subsequently, a colloid dispersion containing the amount of Pt required to get a Pt constant of 1 wt % was contacted with the support in a rotary evaporator at 308 K under stirring for 1 h and then the solvent was removed under vacuum at the same temperature. The samples were dried at 120 ºC for 2 h. Catalysts were labeled as Pt(x)/CNTsZrO_2_ (x is the ligand/Pt molar ratio). The catalyst Pt(0)/CNTsZrO_2_ was prepared by impregnation of chloroplatinic acid in CNTsZrO_2_ followed by calcination at 300 ºC and reduction in hydrogen at 500 ºC.

### 3.4. Characterization

Nitrogen adsorption at 77 K was measured in a Micromeritics ASAP 2010 apparatus. TEM micrographs were obtained in a Jeol Model JEM-1200 EXII System, X-ray diffraction studies were performed in a Rigaku diffractometer.

### 3.5. Hydrogenation reaction

The hydrogenation of 1-phenyl-1,2-propanedione was studied at 298 K and 40 bar of H_2_ with 0.01 mol L^-1^ of substrate, 100 mg of catalyst and 50 mL of cyclohexane as solvent in a 200 mL stainless steel batch reactor . The analysis of reactants and products was followed by a GC-MS device (Shimadzu GCMS-QP5050), using a chiral β-Dex 225, 30 m column (Supelco) and helium as carrier gas.

## 4. Conclusions

The results obtained in the hydrogenation of 1-phenyl-1,2-propanodione on Pt/CNTsZrO_2_ catalysts show that the amount of chiral modifier present on the metallic sites significantly affects catalytic activity, enantioselectivity and the asymmetric hydrogenation reaction. The catalysts were completely regioselective. Thus, only the hydrogenation of the C=O bond located close to the aromatic ring took place, and preferentially led to the corresponding *R*-hydroxyketone. 
